# Profile of the contact tests standard and cosmetic of the Hospital Público do Servidor Estadual de São Paulo

**DOI:** 10.1186/1939-4551-8-S1-A193

**Published:** 2015-04-08

**Authors:** Tatiana Mercuri De Campos, Fatima Rodrigues Fernandes, Débora Nakatani Lopes, Daniele De Sena Brisotto, Camila Campos Teixeira, Barbara Goncalves Da Silva, Wilson Aun, João Ferreira Mello, Maria Elisa Andrade

**Affiliations:** 1 Hospital Do Servidor Público Estadual De São Paulo, Brazil; 2 Department of Allergy and Immunology Francisco Morato De Oliveira Public Servants State Hospital, Brazil

## Background

To evaluate the profile of reactivity of contact tests standard and cosmetics, identify the substances more prevalent and analyze the interaction between them.

## Methods

Retrospective analysis of contact test results, performed with the standard contact test batteries (30 substances) and cosmetics (10 substances) in patients with cutaneous symptoms suggestive of contact dermatitis obtained through database division of Allergy and Immunology of the HSPE- IAMSPE in the period January to December 2013. The contact test is recommended by Brazilian Study Group Contact Dermatitis (GBEDC). It was performed reading after 48 and 96 hours, as international criteria Contact Dermatitis Reserch Group (ICDRG,). Statistical analysis by the test of equality of proportions.

## Results

A total of 749 patients were tested, 588 (78.5 %) women and 161 (21.5 %) men. Of these, 481 (64,21 %) had positive result in reading of 96h. Of these, 68 (9.1 %) were positive in the readings of 48 and 96 h and 413 (55.1 %) only 96h.

A total of 29.960 substances were tested (40 substances in each of 749 patients), and 1.043 (3.5 %) were positive. In the positive tests, 720 (69 %) were positive in the reading of 48 and 96h measure, and 323 (31 %) negative at the 48h and positive in 96 h.

Positive tests in the 96h were classified as weak 482 (46.2%), moderate 287 (27.5%) and strong reactor 274 (26,27%). The most prevalent substances were nickel sulfate (32.7%), cobalt chloride (14.2%), thimerosol (12.8%), neomycin (7.8%), formaldehyde (6.8%), potassium dichromate (4%). Regarding the reactivity of nickel sulfate 51% were classified as strong, 31.8% of moderate and 17.1% weak.

Between the positive substances, it was observed that 393 (37.68%) were metal. In relation to patients, 56 (14,2%) reacted to cobalt and nickel simultaneously, 17(4,3%) patients with to potassium dichromate and nickel, 15 (3,8%) potassium dichromate and cobalt and 6 (1,5%) patients responded to the 3 metals.

## Conclusions

As the literature review indicates nickel sulfate is most prevalent substance and presenting itself as strong reactor mostly, what is significant and there is a strong positive association between metals and it was demonstrated in the present study.

